# De Novo Assembly Discovered Novel Structures in Genome of Plastids and Revealed Divergent Inverted Repeats in *Mammillaria* (Cactaceae, Caryophyllales)

**DOI:** 10.3390/plants8100392

**Published:** 2019-10-01

**Authors:** Sofía Solórzano, Delil A. Chincoya, Alejandro Sanchez-Flores, Karel Estrada, Clara E. Díaz-Velásquez, Antonio González-Rodríguez, Felipe Vaca-Paniagua, Patricia Dávila, Salvador Arias

**Affiliations:** 1 Laboratorio de Ecología Molecular y Evolución, UBIPRO, FES Iztacala, Universidad Nacional Autónoma de México, Avenida de los Barrios 1, Los Reyes Iztacala, Tlalnepantla de Baz 54090, Estado de México, Mexico; dela@comunidad.unam.mx; 2 Unidad Universitaria de Secuenciación Masiva y Bioinformática, Instituto de Biotecnología, Universidad Nacional Autónoma de México, Avenida Universidad 2001, Chamilpa, Cuernavaca 62250, Mexico; karel@ibt.unam.mx; 3 Laboratorio Nacional en Salud: Diagnóstico Molecular y Efecto Ambiental en Enfermedades Crónico-Degenerativas, FES Iztacala, Universidad Nacional Autónoma de México, Los Reyes Iztacala, Tlalnepantla de Baz 54090, Estado de México, Mexico; cdiaz@comunidad.unam.mx (C.E.D.-V.); Felipe.vaca@iztacala.unam.mx (F.V.-P.); 4 Laboratorio de Genética de la Conservación, Instituto de Investigaciones en Ecosistemas y Sustentabilidad, Universidad Nacional Autónoma de México, Antigua carretera a Pátzcuaro 8701, Ex-Hacienda San José La Huerta, Morelia 58190, Michoacán, Mexico; 5 Subdirección de Investigación Básica, Instituto Nacional de Cancerología, Ciudad de México 04510, Mexico; 6 Laboratorio de Recursos Naturales, UBIPRO, FES Iztacala, Universidad Nacional Autónoma de México, Avenida de los Barrios 1, Los Reyes Iztacala, Tlalnepantla de Baz 54090, Estado de México, Mexico; pdavilaa@unam.mx; 7 Jardín Botánico, Instituto de Biología, Universidad Nacional Autónoma de México, Tercer Circuito Exterior, Ciudad Universitaria, Coyoacán, Ciudad de México 04510, Mexico; sarias@ib.unam.mx

**Keywords:** divergent inverted repeats, short-globose cacti, novel gene rearrangements, pseudogenization

## Abstract

The complete sequence of chloroplast genome (cpDNA) has been documented for single large columnar species of Cactaceae, lacking inverted repeats (IRs). We sequenced cpDNA for seven species of the short-globose cacti of *Mammillaria* and de novo assembly revealed three novel structures in land plants. These structures have a large single copy (LSC) that is 2.5 to 10 times larger than the small single copy (SSC), and two IRs that contain strong differences in length and gene composition. Structure 1 is distinguished by short IRs of <1 kb composed by *rpl23-trnI-CAU-ycf2*; with a total length of 110,189 bp and 113 genes. In structure 2, each IR is approximately 7.2 kb and is composed of 11 genes and one Intergenic Spacer-(*psbK-trnQ*)-*trnQ-UUG-rps16-trnK-UUU-matK-trnK-UUU-psbA-trnH-GUG-rpl2-rpl23-trnI-CAU-ycf2*; with a total size of 116,175 bp and 120 genes. Structure 3 has divergent IRs of approximately 14.1 kb, where IRA is composed of 20 genes: *psbA-trnH-GUG-rpl23-trnI-CAU-ycf2-ndhB-rps7-rps12-trnV-GAC-rrn16-ycf68-trnI-GAU-trnA-AGC-rrn23-rrn4.5-rrn5-trnR-ACG-trnN-GUU-ndhF-rpl32*; and IRB is identical to the IRA, but lacks *rpl23*. This structure has 131 genes and, by pseudogenization, it is shown to have the shortest cpDNA, of just 107,343 bp. Our findings show that *Mammillaria* bears an unusual structural diversity of cpDNA, which supports the elucidation of the evolutionary processes involved in cacti lineages.

## 1. Introduction

A new era in the study of evolutionary processes of chloroplasts and their genomes has arisen with the advent of massive sequencing [1]. Huge advances have been documented since 1883, when Schimper postulated an endosymbiotic cyanobacterial origin of these organelles [2]. More recently, many studies have focused on determining the cyanobacterial origin of the DNA molecule contained in chloroplasts [1,3,4]. Using comparative genomics, DNA sequences of complete genomes of contemporary cyanobacteria, algae, and plants have been analyzed, leading to the discovery that the chloroplast genome encompasses structural changes with significant evolutionary information. Thus, in comparison to cyanobacteria and algae, a significant reduction in the total length and in the number of genes has been documented in land plants [5]. However, many genes lacking in the chloroplast genome have migrated to nuclear or mitochondrial genomes [6], which indicates a complex functional relationship among the three genomes contained in plants. In addition, in plants, the chloroplast genome has a hybrid transcriptional process, which denotes the evolutionary transition from a prokaryotic form to a eukaryotic form. Accordingly, most encoding regions are regulated in operons which are transcribed into polycistronic units, as occurs in contemporary cyanobacteria [7]. Additionally, typical eukaryotic transcriptional regulation was documented in nearly 60 promoters for encoding regions and their transfer RNAs [8].

Comparisons within land plants have concluded that the differences in the total length of the complete chloroplast genome (cpDNA) are caused by lengthening or shortening of genes and not by a significant gain/loss of them [5,9]. Flowering land plants tend to have a total of 120 genes; of these, nearly 80 are encoding genes, 30 are tRNAs, and four are rRNAs [9]. In angiosperms, these genes are not randomly distributed along the entire molecule of DNA of the chloroplasts, but instead determine a recognizable structure in the cpDNA. Moreover, in most angiosperms, this cpDNA is sectioned into four regions, which are distinguished by their length and trend in gene composition. The largest single copy (LSC) contains most of the encoding genes directly related to photosystems I and II, ATP synthases, proteins of cytochrome b/f complex, DNA depending on RNA polymerases, and proteins which tend to have a single encoding gene: ribulose bisphosphate carboxylase large chain, maturase K, envelope membrane protein, acetyl coenzyme carboxylase, transcriptional initiation factor, as well as most of proteins of small and large subunits of the ribosome. The small single copy (SSC) often contains dehydrogenase subunits and open reading frames. This copy typically shows the highest mutation rates. The SSC is often flanked by two inverted repeats (IRA and IRB), which vary in gene composition and length [1]. In these plants, the IRs typically contains four ribosomal RNA subunits (*4.5S*, *5S*, *16S*, and *23S*) and five transfer RNA subunits (*trnA-UGC, trnI-GAU, trnN-GUU, trnR-ACG*, and *trnV-GAC*). In addition, the IRs exhibit lower mutation rates than the SSC [10]. Currently, nearly 500 cpDNA have been sequenced for the land plant group, showing that IRs are the main source of structural variation by relative expansion, contraction, and gene rearrangement. However, between IRA and IRB within the same genome, there are no differences in gene arrangement and composition, and in only a few cases do they have low divergence in the DNA sequence [9,10].

In angiosperms, although IRs are commonly present, they are absent in some taxa. Around 95% of legume species of the subfamily Papilionoideae (order Fabales) lack IRs, which has been interpreted as a novel evolutionary change that appeared in a common ancestor and, eventually, was inherited by its descendants, whereas other legume species of this order have IRs [11]. Recently, the lack of IRs was documented in two species of large columnar cacti of Cactoideae (Cactaceae, Caryophyllales) of the tribe Echinocereeae. The loss of IRs in the saguaro (*Carnegiea gigantea*) was interpreted as a novel structural change [12]. In addition, we have verified that the cpDNA of *Pachycereus schottii*, which has recently been directly submitted to the GenBank database, also lacks IRs (uploaded with its synonym *Lophocereus schottii*, NCBI, NC_041727.1). In contrast, in all other species currently sequenced in Caryophyllales have been shown to have IRs [13]. At the infrageneric level, contrasting results have been documented and it is not a rule that all members of a certain genus show identical cpDNA structure. For example, in 13 species of *Camellia* (Theaceae, Ericales), identical structure of cpDNA and low divergence of DNA sequences were documented [14]. A similar result was obtained for seven species of *Silene* (Caryophyllaceae, Caryophyllales), with identical cpDNA structure and only a small gain/loss of genes among them being documented [15,16]. In contrast, unusual results have been obtained for 17 species of *Erodium* (Geraniaceae, Geraniales), which showed deep and strong structural changes, such as expansion and contraction of IRs or even the absence of IRs, and substantial gene rearrangements in the LSC [17]. Thus, data based on characterizations of the structures of complete chloroplast genomes are necessary, as they might reveal novel unexpected results that may help to clarify evolutionary processes in plants.

In this study, we focused on cacti species of the short-globose genus *Mammillaria* (Cactoideae, tribe Cacteae). *Mammillaria* is relevant, in terms of biodiversity, due to its high species richness (163–232) [18] in the Cactaceae. A total of 192 species and subspecies of *Mammillaria* are listed in the Red List of Threatened Species of International Union for Conservation of Nature [19]. For the species of this genus, non-fully resolved phylogenies were obtained from DNA sequences of the r*pl16* intron and psbA-trnH intergenic spacer regions of the chloroplast [20]. The increment of plastid molecular markers (*rpl16, trnK,* and *rpoC1* introns, and *trnK-psbA, rpl20-rps12, trnL-trnF,* and *trnT-trnL* intergenic spacers) did not resolve the relationships among species of *Mammillaria*, nor of species from closer genera (i.e., *Coryphantha, Escobaria, Neolloydia, Ortegocactus,* and *Pelecyphora*). These unresolved evolutionary relationships have been attributed to the recent origin of Cactaceae (e.g., [21,22]), estimated at 35 million years ago [23]. Currently, morphological characteristics have been used to postulate the taxonomic limits among species of *Mammillaria* and of those in close cacti genera [18]. However, these characters are ambiguous and often do not accomplish a robust taxonomically resolved separation [20,21].

In this study, we de novo assembled the complete chloroplast genome of seven species in this genus, in order to utilize these genomes as reference for *Mammillaria*. A second objective was to identify putative structural characteristics of the cpDNA of *Mammillaria*, by comparing with the complete chloroplast genomes, which have been documented for other cacti species and other Caryophyllales. In addition, we discuss whether the structural differences of the cpDNA discovered in *Mammillaria* may serve to resolve the evolutionary and taxonomic pendants of this genus. As structural differences have been documented at the subfamily level, we expect the cpDNA of *Mammillaria* (Cacteae) to differ from those of the large columnar cacti (Echinocereeae); however, among species of *Mammillaria*, structural differences in cpDNA are not expected by its recent divergence.

## 2. Results 

### 2.1. Gene Composition and Length Variation in Three Novel cpDNA Structures Identified in Mammillaria

**Figure 1:**
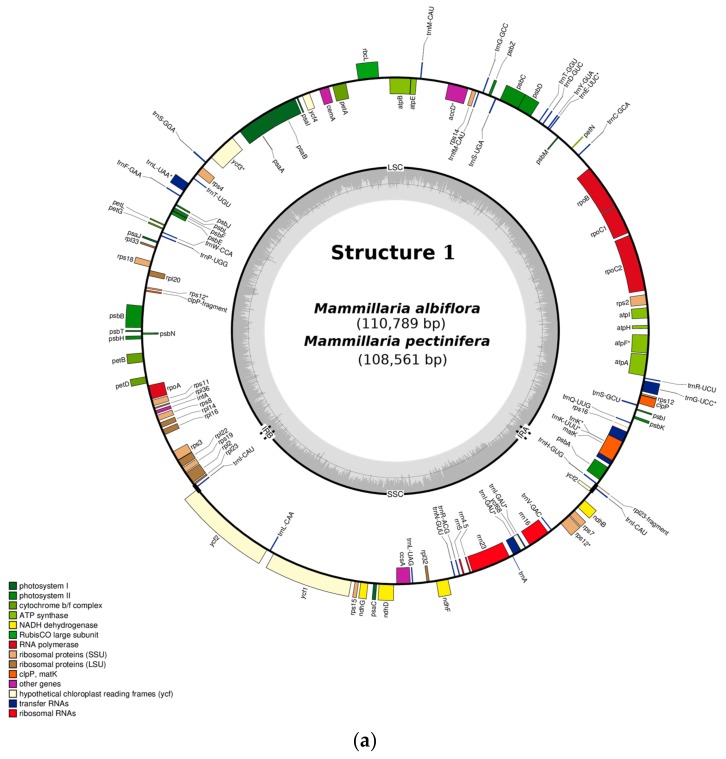

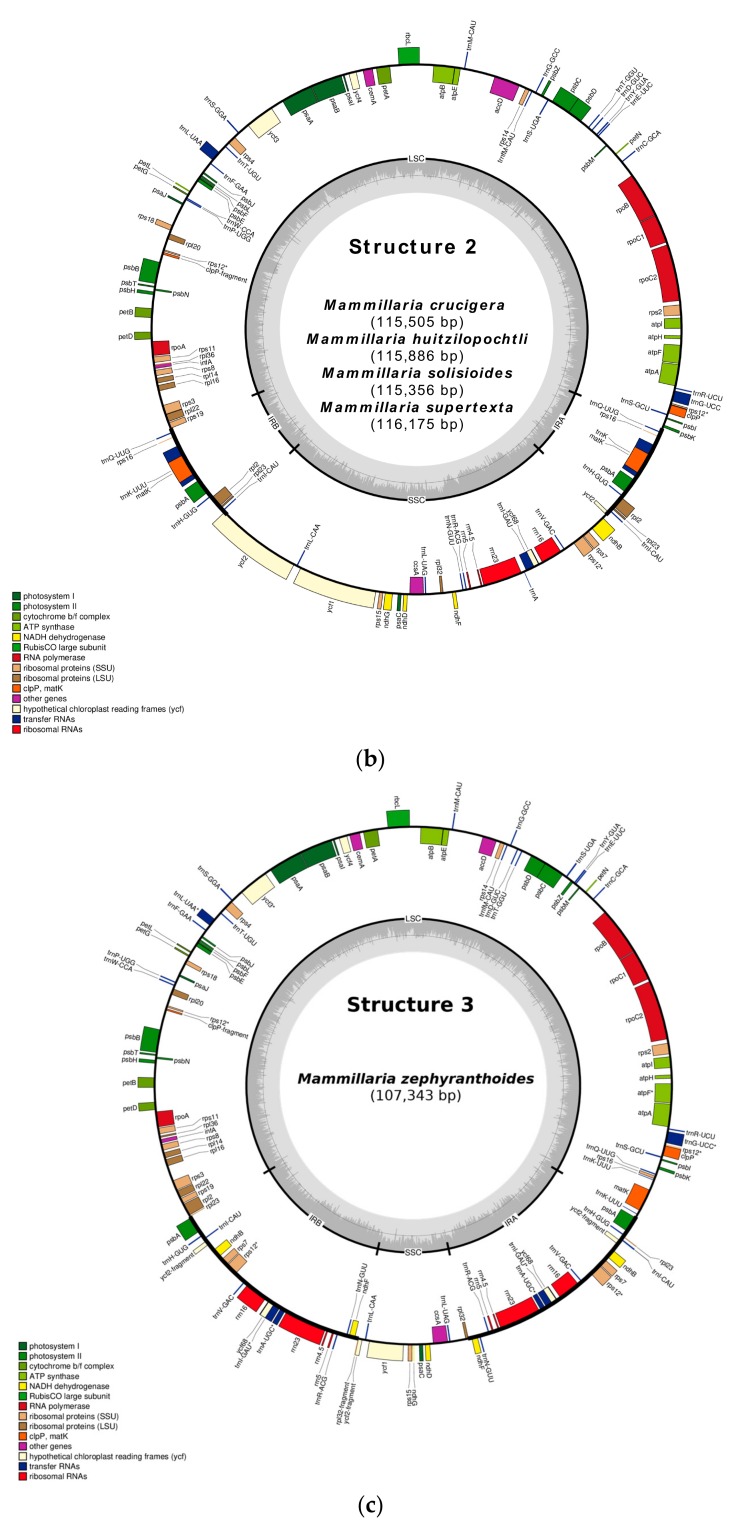
Three different structures found in the complete chloroplast genome of *Mammillaria*: (**a**) structure 1, (**b**) structure 2, and (**c**) structure 3. In structure 1, the *rpl2* gene is flanking IRB in *M. albiflora* and IRA in *M. pectinifera*. Gene *rpl33* was lost in *M. supertexta* of structure 2 and in *M. zephyranthoides* of structure 3. The genomes are displayed circularly, and IRA and IRB correspond to duplicated blocks of regions; starting from the top of the circle, the IRA is the one that appears first in clockwise.

**Figure 2:**
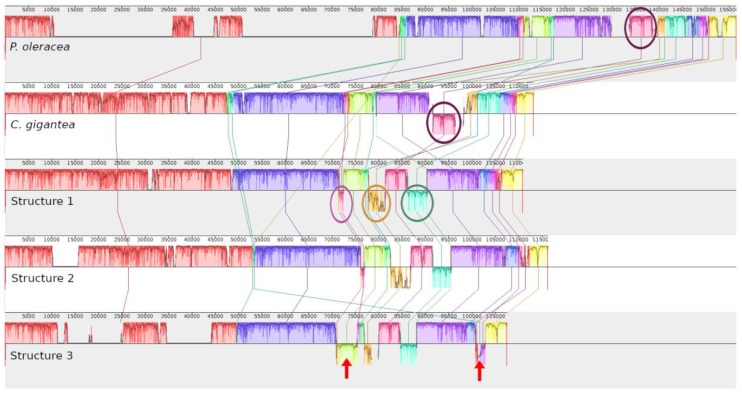
MAUVE graphic of five structural alignments of complete chloroplast genomes. The upper graph corresponds to caryophyllid *P. oleracea* (Portulacaceae); below that, the large giant columnar cactus, *C. gigantea*; and the last three graphs are the three structures documented in *Mammillaria*. Relative inverted DNA sequences are drawn above/below of the horizontal line; identical genes are in the same color. *P. oleracea* has a larger genome than any species of Cactaceae. Discarding the IRs that are recorded in *Mammillaria* and *P. oleracea,* but not in *C. gigantea*, the cpDNA structure of *P. oleracea* is more similar in structure to *C. gigantea* than to *Mammillaria*. Between *C. gigantea* and *P. oleracea*, a single large block of inverted genes (encircled) corresponding to *atpB* and *atpE* is shown. This block of genes in *Mammillaria* has identical orientation to *P. oleracea*. In *Mammillaria*, many other novel gene rearrangements, which are absent in the other two-caryophyllid taxa, were documented. Additionally, structure 3 has two blocks of inverted genes (described in detail in Figure 3b), with respect to structures 1 and 2. These two blocks of genes are indicated with arrows and have identical orientation in *C. gigantea, P. oleracea*, and structures 1 and 2 of *Mammillaria*.

**Figure 3:**
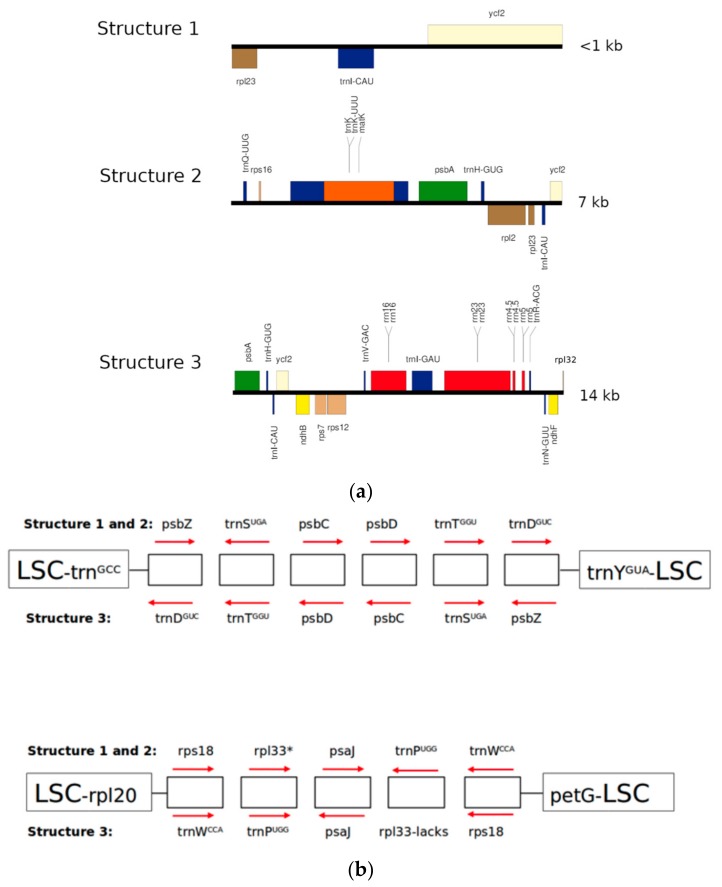
(**a**) Comparison of length and gene composition of IRs in the three structures documented for the complete chloroplast genomes of *Mammillaria*. The two IRs of structure 3 diverge in *rpl23*; its location in IRA is denoted with an asterisk. (**b**) Blocks of genes rearranged at the LSC. These genes are inverted and reoriented in structures 1 and 2, with respect to structure 3. The direction of the row indicates the orientation of transcription, to the left in sense of clockwise and to the right, counter-clockwise. The large squares indicate the genes of LSC that flank these two rearrangements. The asterisk in *rpl33* (bottom figure) indicates that, in *M. supertexta* of structure 2 and in species of structure 3, this gene was lost.

De novo assembly revealed three structures of cpDNA in *Mammillaria* (Figure 1). Structure 1 was present in *M. albiflora* and *M. pectinifera* (Figure 1a), structure 2 in *M. crucigera, M. huitzilopochtli, M. solisioides,* and *M. supertexta* (Figure 1b), and structure 3 in *M. zephyranthoides* (Figure 1c). These structures had a quadripartite partition, into LSC, SSC, and two IRs (Figure 1). We identified unexpected and strong structural differences in gene composition and length among the three structures (Figure 2 and Figure 3a). In addition, structure 3 (*M. zephyranthoides*) had divergent IRs; meanwhile, the rest of the species had identical gene composition in their IRs (Figure 1).

Variation between species in the total length and number of genes of cpDNA were documented (Table 1 and Table 2). The cpDNA of *Mammillaria* ranged from 107,343 bp (*M. zephyranthoides*) to 116,175 bp (*M. supertexta*) (Table 1). The relative length of LSC represented approximately 62% of the genome in the six species (*M. albiflora, M. crucigera, M. huitzilopochtli, M. pectinifera, M. solisioides,* and *M. supertexta*) (Table 1). Moreover, the LSC was nearly 2.5 times larger than SSC, which represented 25.35–28% of the total genome length (Table 1). In contrast, in *M. zephyranthoides*, the LSC was longer (68% of the cpDNA length), being 10 times larger than its respective SSC, whose length only reached 7%. The shortening of SSC in *M. zephyranthoides* was due to the lengthening of the IRs, which had nearly 14 kb, and to the strong reduction of the genes *ycf1* and *ycf2* to <1 kb; meanwhile, in the other species of *Mammillaria*, these genes were >6 kb (Figure 1, Table 1). 

We identified additional different types of gene rearrangements at the LSC (Figure 3b). The first was a type of rearrangement involving blocks of genes, which were inverted but maintained identical order (Figure 3b); there was also a second type involving a single gene with two variants: a) the single gene did not change its relative location, but its orientation was inverted (*trnF-GAA*); b) the single gene changed in location, but it maintained its orientation (*rpl2*). The single gene *trnF-GAA* had identical orientation in the species of structure 1 (*M. albiflora* and *M. pectinifera*), structure 3 (*M. zephyranthoides*), and *M. solisioides* of structure 2, but was inverted in the other three species of structure 2 (*M. crucigera, M. huitzilopochtli,* and *M. supertexta*). In the structure 1 species, *rpl2* flanked the IRA in *M. pectinifera*, whereas, in *M. albiflora*, it flanked the IRB. In addition, in *M. supertexta* (structure 2) and *M. zephyranthoides* (structure 3), *rpl33* was lost, but it was present in the other five species of *Mammillaria* as pseudogene except in *M. albiflora* (Figure 1, Table 2). 

On the other hand, comparison of the complete genomes of the seven species showed similar percentages of types of genes. In the three structures, the highest percentage of genes corresponded to tRNAs (26%), where each of the sets of *rps* and *psb* represented 13% of the genes. Another similarity found among species was that, in the LSC, large blocks of concatenated genes maintained identical gene compositions and arrangements (Figure 1 and Figure 2). Most of the concatenated genes correspond to the encoding genes of photosystems I and II. In addition, the *rbcL* gene, units of the cytochrome b/f complex, and genes of the DNA-dependent RNA polymerase (Table 2) were identical in number, location, and arrangement (Figure 1). Comparisons of LSC and SSC showed that structures 1 and 2 had more mutual similarities than either had with structure 3 (Table 2). In the SSC, the seven species maintained identical order and orientation in *ycf2-trnL-CAA-ycf1*; although, in structure 3, they were shorter by pseudogenization (Figure 1). 

In addition, in the seven species pseudogenization was identified in the NADH dehydrogenase-like (NDH) complex of plastid genes (*ndh* genes, hereafter). Of this family of genes, only four subunits of the suite of dehydrogenase genes (B, D, F, and G) were recorded in the seven species. The *ndh* subunits B, D, and F were pseudogenes in the seven species, and subunit G was pseudogene only in *M. pectinifera, M. solisioides* and *M. zephyranthoides*. In addition, *ycf68* was a pseudogene in all seven species, and *ycf4* was pseudogene in three species except in *M. albiflora*, *M. huitzilopochtli*, *M. supertexta* and *M. zephyranthoides* (Table 2).

### 2.2. Structure 1: Shortest IRs, Composed of Three Genes, rpl23-trnI-CAU-ycf2

This structure was distinguished by two short IRs (of <1 kb) composed by *rpl23-trnI-CAU-ycf2.* Of this *ycf2*, a total of 265 bases of its 5’ extreme are inserted in the IRs, which were identical in DNA sequence and gene composition. This structure 1 was found only in two species of *Krainzia* subgenus, *M. albiflora*, and *M. pectinifera* (Figure 1, Table 1). The genome of *M. albiflora* was larger (110,789 bp, Table 1) than the one of *M. pectinifera*. In this structure, both IRs had length 1348 bp (1.22% of the total genome). The three genes represented 5.3% of the total genes (113). These two species had an identical number of total genes; most of which (26.6%) were represented by tRNAs (Figure 1a, Table 1). The LSC covered the largest proportion of the DNA sequence (72.6%, Table 1) and the largest number of genes (82) (Table 2). However, *rpl2* gene is flaking IRA in *M. pectinifera* but in *M. albiflora* is flanking IRB that is the identical location of the species of structures 2 and 3. 

### 2.3. Structure 2: IRs Composed by an Unusual Complete Battery of 11 Concatenated Genes and One Identical Intergenic Spacer.

Structure 2 was found in three species of the subgenus *Mammillaria* (*M. crucigera*, *M. Huitzilopochtli*, and *M. supertexta*) and one of the subgenus *Krainzia* (*M. solisioides*). In all of these, the two IRs were formed by genes usually located at the LSC, and were flanked by the DNA sequence of an identical intergenic spacer sequence (IGS) that is from psbk to trnQ; however, IRB was flanked by *rps19*, whereas IRA was flanked by *psbK* (Figure 1). The complete composition of this structure consisted of this IGS and 11 genes: IGS (*psbK-trnQ*)*-trnQ-UUG-rps16-trnK-UUU-matK-trnK-UUU-psbA-trnH-GUG-rpl2-rpl23-trnI-CAU-ycf2*. The four species with structure 2 had identical IRs, with respect to gene composition and DNA sequence. However, there were differences between species, in terms of the total genome length, number of genes, and length of each of the four quadripartite regions (Table 1). The *rpl33* gene was found in three species, but was absent in *M. supertexta*. Consequently, the total number of genes differed among the structure 2 species (Table 1 and Table 2). Although they showed differences, the four species had similar percentages in the relative proportions of genes represented in IRs, LSC, and SSC, as well as in the percentages of gene types in the overall genome (Table 1 and Table 2). Using *M. supertexta*, as a reference is important, as it had the largest genome in structure 2, showing 63% (75) of genes located at the LSC and 20.2% (24) at the SSC. In addition, each IR comprised 6.24% of the genes (11 genes and one IGS). In structure 2, the complete gene of maturase K gen (*matK*) is nested with *trnK-UUU* introns and inserted into the IRs. In this structure, 28.20% were tRNAs, followed by 13.67% in each of the suites of *rps* and *psb* subunits, *rpl* subunits represent 8.40%. Finally, the DNA regions that were represented by only one gene (0.85%) were *accD*, *ccsA*, *cemA*, *infA*, and *rbcL* (Table 2).

### 2.4. Structure 3: Largest and Divergent IRs in Which Four Ribosomal Units are Included.

Structure 3 was only recorded in *M. zephyranthoides* (Figure 1c). This genome had a length of 107,343 bp and 131 genes (Table 1). The LSC covered 62% of the total number of genes (81), the SSC had only 7 genes (5.7%), and both IRs had 43 genes (32.8%). In addition, each IR was approximately 14.1 kb in length and was comprised of genes typically located at the SSC. The IRA was composed of psbA-trnH-GUG-rpl23*-trnI-CAU-ycf2partial-ndhB-rps7-rps12-trnV-GAC-rrn16-ycf68-trnI-GAU-trnA-AGC-rrn23-rrn4.5-rrn5-trnR-ACG-trnN-GUU-ndhF-rpl32. The *rpl23* is marked with an asterisk because it is absent in the IRB, thus the IRB was identical in gene composition and arrangements but was divergent. Structure 3 is distinguished by the ancestral presence of four rRNA subunits (4.5, 5, 16, and 23) in the IRs. 

Pseudogenization in structure 3 was clearly identified, in that *ycf2* and *ycf1* showed incomplete DNA sequences. The former was truncated into three segments. Two of these segments were inserted into the IRS and the third one was at the SSC. These three segments added only to a total of 960 bp. The gene *ycf1* had <1000 bp. Consequently, the shortening of *ycf1* and *ycf2* caused a diminished cpDNA total length (Table 1, Figure 1c). Additionally, this structure added, as pseudogenes, *accd, rps18, rpl23,* and one copy of *rps12*.

## 3. Discussion

The three structures of cpDNA discovered in *Mammillaria* are novel and these have not been recorded in other eukaryote organisms. In addition, the divergent IRs (identified in structure 3) are a novel result for land plants (Embryophyta), only having otherwise been recently discovered in green algae of the order Ignatiales (*Pseudoneochloris*, and *Chamaetrichon*) [24]. These strong arrangements in the cpDNA of *Mammillaria* are notable with respect to the rest of caryophyllids as *P. oleracea* (Portulacaceae) and *C. gigantea* (Cactaceae), and even within *Mammillaria* (Figure 2 and Figure 3). Based on the DNA sequences of chloroplasts and current biogeographic distribution, it was estimated that the suborder Portulacinae, which includes the families Cactaceae and Portulacaceae, diverged in the early Miocene (18.8–33.7 Mya). In particular, for Cactaceae, an origin of 10–19 Mya has been estimated [25]. This last estimation differs from the age of 35 Mya estimated for Cactaceae based on molecular phylogeny [23]. Accordingly, the members of Cactaceae are relatively young in the evolutionary history of Caryophyllales and, thus, the structures of cpDNA found in *Mammillaria* have evolved in a recent diverging process, as none of the three structures have been reported for other, older members of Caryophyllales. 

Our results suggest that the structural reconfigurations of cpDNA within the family Cactaceae have occurred frequently. Particularly, such structural changes have mainly involved genes located at the flanking extremes of the LSC and, secondly, genes located at the SSC. Although deep and strong changes occurred in reconfigurations of the IRs, only few gene rearrangements and loss of genes occurred in the LSC, protecting the large blocks of genes involved in photosynthesis (Figure 2). We conclude that no single type of cpDNA structure characterizes all members of *Mammillaria*; however, the presence of IRs is a marked difference, with respect to the large columnar cacti species that have lost them.

**Figure 4:**
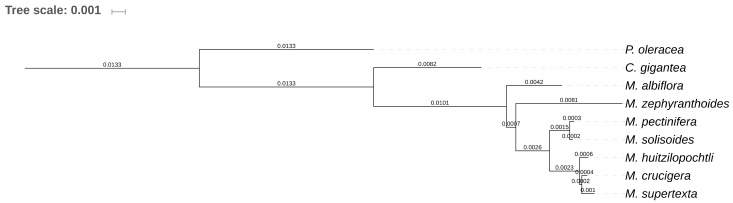
Phylogenetic ML tree obtained for the seven species of *Mammillaria*. The analysis is based on 42 coding regions shared to the two species used as outgroups (*C. gigantea* and *P. oleraceae*).

In addition, our results showed that, among the tribes of the subfamily Cactoideae, there are notable structural differences in the cpDNA. The most evident is that *Mammillaria* (Cacteae) has IRs, as occurs in most of Caryophyllales, which were lost in Echinocereeae. We propose that the presence of IRs is the basal state in the Cactaceae family, as the presence of IRs is common in all members of Caryophyllales currently sequenced. However, we cannot ignore the possibility that IRs may be have been lost and recovered (with a new configuration) in multiple evolutionary events occurring during Cactaceae radiation. The phylogenetic relationships of the seven species based on DNA sequences (Figure 4) showed that *M. albiflora* (structure 1) is the closest species to columnar saguaro, however, *M. pectinifera* (structure 1) is the closest one to *M. solisioides* (structure 2). Unexpectedly, *M. zephyranthoides* (structure 3) occupied a branch that is closer to the three species of series *Supertextae*. These phylogenetic results should be taken with caution, since the number of species is too poor; however, it seems that the evolutionary underlying processes that have operated on the structural changes differ of those operating at the level of DNA sequences.

Our findings showed that, in *Mammillaria*, the concatenated battery *rpl23-trnI-CAU-ycf2* might have a relevant role in the reconfiguration of IRs. Particularly in these genes, it is worthy to highlight the gene *trnI-CAU*, which (along *with trnI-GAU, trnfM-CAU*, and *trnM-CAU*) has been identified, in experimental essays, as one of four essential tRNAs in plastids [26]. In addition, the IRs composed of a single gene have been shown to correspond to *trnI-CAU* (e.g. *Pinus massoniana*) [27]. In this context, we propose that *trnI-CAU* in *Mammillaria* plays a key role in the reconfiguration of IRs, but future studies are needed to verify this.

We found that the IGS *psbK-trnQ* was inserted in both IRs of structure 2, although this IGS at IRB was flanked by *rps19* at the LSC; meanwhile, in the rest of the species, this pair of genes (*psbk* and *trnQ*) was located at the LSC. In addition, the DNA sequence of this IGS was highly conserved, showing 91–97% of identity to the IGS of other Caryophyllales species (e.g., *C. gigantea, Cistanthe longiscaspa, Tallinum paniculatum,* and *P. oleracea*). In addition, we found other highly conserved IGS, which showed 90–98% of identity to the IGS of *trnG-UCC—trnS-GGU* in other members of Caryophyllales. However, in *Mammillaria*, this pair of transfer genes was at the contrary extreme of the LSC, although the DNA sequence of this IGS was found between *rpl20-rps12*. Thus, we support the idea that IGS may have played an important role in the functional processes, in agreement with former studies [28]. Pseudogenization was identified in the seven species of *Mammillaria*, but in structure 3 it was more evident (Figure 1 and Figure 2); particularly by the strong shortening of the genes of the two open reading frames, *ycf2* and *ycf1*, as they had a length of <1 kb whilst, in the other six species of *Mammillaria*, each of these genes had a length of nearly 6–7 kb. The pseudogenization of *ycf1* and *ycf2* indicates a loss of functional activity, which disagrees with the conclusion that these genes are essential for plant survival [29]. In addition, pseudogenization was also identified for all species of *Mammillaria*, with incomplete copies of subunits of *rps* and *rpl* suites, *accD*, as well as, *rps12* and *clpP* duplicated and, evidently, in three dehydrogenase subunits (B, D, and F). These subunits translated to an interrupted sequence of amino acids, which indicates that the functionalities of these genes may have been lost. In addition, seven other subunits of *ndh* genes were completely lost (A, C, E, H, I, J, and K). These subunits in *C. gigantea*, both pseudogenization and the complete loss of *ndh* subunits in cpDNA have also been documented [12]. However, we could not show, for *Mammillaria*, that all of those genes documented as pseudogenes, or entirely lacking in the cpDNA, were not present in nuclear or mitochondrial genomes; in other plants, many genes of the chloroplast have been found in nuclear or mitochondrial genomes [6].

An interesting result, obtained in the SSC of the seven species of *Mammillaria*, *C. gigantea*, and *P. schottii*, is that they all have identical order and orientation of *ycf2-trnL-CAA-ycf1*. This result suggests that the arrangement of these three genes may be a synapomorphy in the subfamily Cactoideae, as it is not present in *Opuntia microdasys*, subfamily Opuntioideae (sequence consulted GenBank: HQ664651.1), nor in the rest of the species of Caryophyllales [13].

Structure 2 was distinguished by the insertion of the *matK* gene into the IRs, which was nested inside of two complete *trnK* introns. The gene *matK* was also documented in the IRs of some species of *Erodium* (Geraniaceae) but was truncated and separated to the two *trnK* introns [17]. In addition, in IRs of *Lamprocapnos spectabilis* (Papaveraceae), complete *matK* nested between the two introns has been documented [30]. It is relevant to point out that *matK* is duplicated in these genomes due to its insertion in IRs. This encoding gene plays a fundamental role in photosynthesis by editing the RNAs of nearly 15 proteins, even though the *trnK* introns were lost [31]. Thus, in species having structure 2, by the formation of IRs, there are two copies of *matK* in the haploid genome of the chloroplast; however, we do not possess sufficient information to discuss the functional consequences of this.

Finally, it is important to point out the following: 1) the three structures of cpDNA of *Mammillaria* are not concordant to the taxonomic subgenera and series levels. Consequently, our results do not support any of the infrageneric classification currently proposed. 2) *Sensu* [18], *M. solisioides* is considered to be a subspecies of *M. pectinifera* (subgenus *Krainzia*, series *Herrerae+Pectiniferae*); however, *M. solisioides* exhibits the cpDNA structure found in the *Supertextae*, subgenus *Mammillaria*. 3) Accordingly, we consider that *M. solisioides* is an independent species, in agreement with Arias et al. [32]. We conclude that the structural analysis of cpDNA can also contribute towards clarifying the taxonomic relationships of *Mammillaria* with other plant species.

## 4. Materials and Methods

### 4.1. Plant Sampling and DNA Extraction

We included seven species of *Mammillaria*, which represent three of the eight subgenera proposed by Hunt [18]. These seven species are listed in the IUCN red list [19]. The three species sampled (*M. crucigera*, *M. huitzilopochtli*, and *M. supertexta*) are classified in the subgenus *Mammillaria*, series *Supertextae.* According to Crozier [21], only the species included in this subgenus represent a natural and monophyletic clade in phylogenies obtained with chloroplast DNA sequences. The other three taxa sampled (*M. albiflora*, *M. pectinifera*, and *M. solisioides)* are of the subgenus *Krainzia*, series *Herrerae+Pectiniferae*. It is important to mention that *M. solisioides* was considered [18] to be a subspecies of *M. pectinifera*. The last sampled species was *M. zephyranthoides*, classified in the subgenus *Dolichothele*. This last species has faced a complex and controversial taxonomic identification, as it has been included in both *Dolichothele* and *Mammillaria*.

Living complete plants for tissue samples were obtained for *M. albiflora*, *M. crucigera*, *M. huitzilopochtli*, *M. pectinifera*, *M. solisioides*, and *M. supertexta*. Small pieces of the surface of green stems were cleaned of spines and areoles to obtain 300 mg samples of tissue, which were treated using a MinuteTM Chloroplast Isolation Kit (Invent Technologies Inc., Eden Prairie, MN, USA), according to the manufacturer’s instructions. The chloroplast extracts were processed with a DNeasy plant minikit (Qiagen, Valencia, CA, USA), in order to obtain enriched chloroplast genomes.

### 4.2. High-Throughput Sequencing and Sanger Verification

Massive sequencing was done with the Illumina platform (Ilumina, San Diego, CA, USA). For each species, genomic libraries were prepared with the Nextera XT kit, according to the manufacturer’s instructions, and sequenced in a MiSeq 2 × 300 cycles. The flanks of IRs and gene rearrangements were PCR verified (Table S1), using the recently assembled cpDNA in our study for the design of specific primers with Primer3 v.4 [33]. These PCR products were sequenced in a 3730×l capillary sequencer (Applied Biosystems, Pleasanton, CA, USA).

### 4.3. Genome Assembly, Annotation, and Structural Alignment

The available assembly genome for the giant columnar cactus species of *Carnegiea gigantea* [12] lacks IRs and, thus, de novo assembly was carried out with NovoPlasty v.2.6.5. [34] and DISCOVAR de novo v.52488 [35]. The scaffolding was carried out with Ragout v.2.0 [36]. The gaps were filled with GARM v.0.7.5 [37] and the circularization of each of the assembly genomes was obtained with Circlator v.1.5.5 [38]. The assemblage of each genome was tested with REAPR v.1.0.18 [39]. The annotation for the seven species was done with GeSeq [40] and the genomes were drawn with OGD [41]. For gene annotation, we used the cpDNA of *C. gigantea* [12] and *Portulaca oleracea* [42]. Annotation of the largest genome found for each of the three different structures (Table 1) was manually curated. The structural alignment of the complete assembly genomes was performed with MAUVE [43]. This program was also used to compare these structures to other species of Caryophyllales (*P. oleracea*, NC_036236.1) and Cactaceae (*C. gigantea*, GCA_002740515.1), in order to emphasize the relevance of our structural findings in the whole chloroplast genome of *Mammillaria*. In order to reconstruct the phylogenetic relationships of the seven species of *Mammillaria*, two species (*C. gigantea* and *P. oleracea*) were used as outgroups. A total of 42 orthologous protein-coding genes (Table S2) shared in these nine species were identified with Prottest [44]. The DNA sequences of these 42 loci were aligned using MAFFT v7.310 [45]. The Akaike Information Criterion (AIC) in JMODELTEST v2.1.10 was used to determine the best-fitting model of nucleotide substitutions [46]. The GTR + G model was used to obtain the phylogenetic tree based on ML in RAXML-HPC v8.2.10 [47] with 1000 replicates.

## Figures and Tables

**Table 1:** Species of *Mammillaria* grouped by the type of the structure identified in the complete chloroplast genome (cpDNA). Within and among structure variation in total length size, the two inverted repeats (IRs), large single copy (LSC), and small single copy (SSC) were detected.

Type of Structure	Total Length	IRs	LSC	SCC	Total Number of Genes	Access Number ^1^
I. Structure 1						
1.1 *M. albiflora*	110789	1348	78380	31061	113	MN517610
1.2. *M. pectinifera*	108561	1544	72273	29744	113	MN519716
II. Structure 2						
1. *M. crucigera*	115505	14522	71565	29418	120	MN517613
2. *M. huitzilopochtli*	115886	14488	71997	29401	120	MN517612
3. *M. solisioides*	115356	14428	71690	29238	120	MN518341
4. *M. supertexta*	116175	14490	72240	29445	119	MN508963
III. Structure 3						
1. *M. zephyranthoides*	107343	28252	71811	7281	131	MN517611

^1^ GeneBank access number of the DNA sequences deposited.

**Table 2:** Variation in structural and functional gene composition in the three structures of cpDNA found in *Mammillaria*. A total of 18 different types of genes were documented, and these are organized alphabetically according to their location in IRs, LSC, and SSC. All the genes located at IRs are duplicated (2X), except the *rpl23*Ψ in structure 3 that lacks in IRB.

Gene Type/Structure	Region	Structure 1	Structure 2	Structure 3
1. Ribosomal RNA (rrn)	SSC	rrn4.5, 5,16, 23	rrn4.5, 5,16, 23	
	IRs			rrn4.5, 5,16, 23 (2X)
2. Transfer RNA (trn)	LSC	trnC^GCA^, trnD^GUC^, trnE^UUC^, trnF^GAA^, trnG^GCC^, trnG^UCC^, trnH^GUG^, trnK^UUU^, trnL^UAA^, trnM^CAU^, trnM^CAU^, trnP^UGG^, trnQ^UUG^, trnR^UCU^, trnS^GGA^, trnS^GGU^, trnS^UGA^, trnT^GGU^, trnT^UGU^, trnY^GUA^	trnC^GCA^, trnD^GUC^, trnE^UUC^, trnF^GAA^, trnG^GCC^, trnG^UCC^, trnL^UAA^, trnM^CAU^, trnfM^CAU^, trnP^UGG^, trnR^UCU^, trnS^GGA^, trnS^GCU^, trnS^UGA^, trnT^GGU^, trnT^UGU^, trnW^CCA^, trnY^GUA^	trnC^GCA^, trnD^GUC^, trnE^UUC^, trnF^GAA^, trnG^GCC^, trnG^UCC^, trnK^UUU^, trnL^UAA^, trnM^CAU^, trnfM^CAU^, trnP^UGG^, trnQ^UUG^, trnR^UCU^, trnS^GGA^, trnS^GCU^, trnS^UGA^, trnT^GGU^, trnT^UGU^, trnW^CCA^, trnY^GUA^
	SSC	trnA-f, trnI^GAU^, trnI^GAU^, trnL^CAA^, trnL^UAG^, trnN^GUU^, trnR^ACG^, trnV^GAG^	trnA-f, trnI^GAU^, trnI^UAG^, trnN^GUU^, trnL^CAA^, trnR^ACG^, trnV^GAG^	trnL^UAG^, trnL^CAA^
	IRs	trnI^CAU^ (2X)	trnH^GUG^,tmI^CAU^, trnK^UUU^, trnQ^UUG^ (2X)	trnA^UGC^, trnH^GUG^, trnI^CAU^, trnI^GAU^, trnN^GUU^, trnR^ACG^, trnV^GAC^ (2X)
3. Proteins of small subunits of the ribosome (rps)	LSC	rps2, 3, 4, 8, 11, 12 (2), 14, 16Ψ, 18Ψ, 19	rps2, 3, 4, 8, 11, 12 (2), 14, 18Ψ, 19	rps2, 3, 4, 8 11, 12, 12Ψ, 14, 16Ψ, 18Ψ, 19
	SSC	rps7, 12, 15	rps7, 12, 15	rps15
	IRs		rps16Ψ (2X)	rps7, 12, (2X)
4. Proteins of large subunits of the ribosome (rpl)	LSC	rpl2, 14, 16, 20, 22, 33Ψ, 36Ψ	rpl14, 16Ψ, 20, 22,33Ψ*, 36Ψ	rpl2, 14, 16Ψ, 20, 22, 23Ψ, 36
	SSC	rpl32	rpl32	
	IRs	rpl23Ψ (2X)	rpl2, 23Ψ (2X)	rpl32 (2X), 23Ψ (IRA)
5. DNA dependent RNA polymerase (rpo)	LSC	rpoA, B, C1, C2	rpoA, B, C1, C2	rpoA, B, C1, C2,
6. NADH dehydrogenase (ndh)	SSC	ndhBΨ, DΨ, FΨ, GΨ***	ndhBΨ, DΨ, FΨ, G***	
	IRs			ndhBΨ, DΨ, FΨ, GΨ (2X)
7. Photosystem I (psa)	LSC	psaA, B, I, J	psaA, B, I, J	psaA, B, I, J
	SSC	psaC	psaC	psaC
8. Photosystem II (psb)	LSC	psbA, B, C, D, E, F, H, I, J, K, L, M, N, T, Z	psbB, C, D, E, F, H, I, J, K, L, M, N, T, Z	psbB, C, D, E, F, H, I, J, L, K, M, N T, Z
	IRs		psbA (2X)	psbA (2X)
9. Cytochrome b/f complex (pet)	LSC	petA, B, D, G, L, N	petA, B, D, G, L, N	petA, B, D, G, L, N
10. ATP synthase (atp)	LSC	atpA, B, E, F, H, I	atpA, B, E, F, H, I	atpA, B, E, F, H, I
11. Rubisco (rbc)	LSC	rbcL	rbcL	rbcL
12. Maturase K	LSC	matK		matK
	IRs		matK (2X)	
13. Protease (clp)	LSC	clpPΨ, clpP	clpPΨ, clpP	clpPΨ, clpP
14. Envelope membrane protein (cem)	LSC	cemA	cemA	cemA
15. Subunit of acetil-CoA-carboxylase (acc)	LSC	accDΨ	accDΨ	accDΨ
16. c-type cytochrome synthesis (ccs)	SSC	ccsA	**SSC**: ccsA	**SSC**: ccsA
17. Translational initiation factor (inf)	LSC	infA	infA	infA
18. Hypothetical chloroplast reading frames (ycf)	LSC	ycf3, ycf4Ψ	ycf3, ycf4Ψ**	ycf3, ycf4
	SSC	ycf1, ycf2, ycf68Ψ	ycf1, ycf2, ycf68Ψ	ycf1Ψ, ycf2Ψ
	IRs	ycf2-p (2X)	ycf2-p (2X)	ycf2Ψ, ycf68Ψ (2X)

Ψ indicates a pseudogene. The note “-p” indicates that a partial DNA sequence of a gene is inserted in the two IRs. * indicates that *rpl33* lacks in *M. supertexta* but it is present in the other three species of this structure. In addition, this gen is pseudogene in all species except in *M. albiflora*. ** indicates that is a pseudogene in *M. crucigera* and *M. solisioides* of structure 2. *** indicates that it is pseudogene only in *M. solisioides* of structure 2 and *M. pectinifera* of structure 1.

## Supplementary Materials

The following are available online at https://www.mdpi.com/2223-7747/8/10/392/s1, Table S1: Primer sequences designed for PCR verifications for flanking adjacent sequences of IRs; and gene rearrangements located at LSC in three genomes structures of *Mammillaria.* In the last column the temperature used to amplify each locus for all species. Primer design was based on the DNA sequences of *M. albiflora* (structure 1), *M. supertexta* (structure 2) and *M. zephyranthoides* (structure 3); Table S2. List of the total of 42 coding loci were used to obtain phylogenetic relationships of the seven species of *Mammillaria* studied and the two species used as outgroups *Carnegiea gigantea and Portulaca oleraceae*.

## References

[1] Daniell H., Lin C.S., Yu M., Chang W.J. (2016). Chloroplast genomes: Diversity, evolution, and applications in genetic engineering. Genome Biol..

[2] Morden C., Delwiche C., Kuhsel M., Palmer D. (1992). Gene phylogenies and the endosymbiotic origin of plastids. Biosystems.

[3] Cavalier-Smith T. (1982). The origins of plastids. Biol. J. Linn. Soc..

[4] Lemieux C., Otis C., Turnel M. (2000). Ancestral chloroplast genome in *Mesostigma viride* reveals an early branch of green plant evolution. Nature.

[5] Xiao-Ming Z., Junrui W., Li F., Sha L., Hongbo P., Lan Q., Jing L., Yan S., Weihua Q., Lifang Z. (2017). Inferring the evolutionary mechanism of the chloroplast genome size by comparing whole-chloroplast genome sequences in seed plants. Sci. Rep..

[6] Thorsness P.E., Weber E.R. (1996). Escape and migration of nucleic acids between chloroplasts, mitochondria, and the nucleus. Int. Rev. Cytol..

[7] Yagi Y., Shiina T. (2014). Recent advances in the study of chloroplast gene expression and its evolution. Front. Plant Sci..

[8] Kung S.D., Lin C.M. (1985). Chloroplast promoters from higher plants. Nucleic Acids Res..

[9] Mower J.P., Vickrey T.L., Chaw S.M., Jansen R.K. (2018). Advances in Botanical Research Plastid Genome Evolution.

[10] Zhu A., Guo W., Sakski G., Weishu F., Mover J.P. (2016). Evolutionary dynamics of the plastid inverted repeat: The effects of expansion, contraction, and loss of substitution rates. New Phytol..

[11] Lavin M., Doyle J.J., Palmer J.D. (1990). Evolutionary significance of the loss of the chloroplast-DNA Inverted Repeat in the Leguminosae Subfamily Papilionoideae. Evolution.

[12] Sanderson M.J., Copetti D., Búrquez A., Bustamante E., Charboneau J.L.M., Eguiarte L., Kumar S., Lee H.O., McMahon M., Steele K. (2015). Exceptional reduction of the plastid genome of saguaro cactus (*Carnegiea gigantea*). Am. J. Bot..

[13] Yao G., Jin J.J., Li H.T., Yang J.B., Mandala V.S., Croley M., Mostow R., Douglas N.A., Chase M.W., Christenhusz M.J.M. (2019). Plastid phylogenomic insights into the evolution of Caryophyllales. Mol. Phylogenet. Evol..

[14] Huang H., Shi C., Liu Y., Mao S.Y., Gao L.Z. (2014). Thirteen *Camellia* chloroplast genome sequences determined by high-throughput sequencing: Genome structure and phylogenetic relationships. BMC Evol. Biol..

[15] Wu Z., Tembrock L.R. (2015). Two complete chloroplast genomes of white campion (*Silene latifolia*) from male and female individuals. Mitochondrial DNA Part A.

[16] Kang J.S., Lee B.Y., Kwak M. (2017). The complete chloroplast genome sequences of *Lychnis wilfordii* and *Silene capitata* and comparative analyses with other Caryophyllaceae genomes. PLoS ONE.

[17] Blazier J.C., Jansen R.K., Mower J.P., Govindu M., Zhang J., Weng M.L., Ruhlman T.A. (2016). Variable presence of the inverted repeat and plastome stability in *Erodium*. Ann. Bot..

[18] Hunt D., Taylor N., Charles G. (2006). The New Cactus Lexicon.

[19] IUCN International Union for Conservation of Nature Consulted for Mammillaria genus. https://www.iucnredlist.org/search/list?query=Mammillaria&searchType=species.

[20] Butterworth C.A., Wallace R.S. (2004). Phylogenetic studies of *Mammillaria* (Cactaceae)—Insights from chloroplast sequence variation and hypothesis testing using the parametric bootstrap. Am. J. Bot..

[21] Crozier B.S. (2005). Systematics of Cactaceae Juss: Phylogeny, cpDNA Evolution, and Classification, with Emphasis on the Genus *Mammillaria* Haw. Ph.D. Thesis.

[22] Hernández-Hernández T., Hernández H.M., De Nova J.A., Puente R., Eguiarte L., Magallón S. (2011). Phylogenetic relationships and evolution of growth form in Cactaceae (Caryophyllales, Eudicotyledoneae). Am. J. Bot..

[23] Arakaki M., Christin P.A., Nyffeler R., Lendel A., Eggli U., Ogburn M., Spriggs E., Moore M.J., Edwards E.J. (2011). Contemporaneous and recent radiations of the world’s major succulent plant lineages. Proc. Natl. Acad. Sci. USA.

[24] Turmel M., Otis C., Lemieux C. (2017). Divergent copies of the large inverted repeat in the chloroplast genomes of ulvophycean green algae. Sci. Rep..

[25] Ocampo G., Columbus J.T. (2010). Molecular phylogenetics of suborder Cactineae (Caryophyllales), including insight into photosynthetic diversification and historical biogeography. Am. J. Bot..

[26] Alkatib S., Fleischmann T.T., Scharff L.B., Bock R. (2012). Evolutionary constraints on the plastid tRNA set decoding methionine and isoleucine. Nucleic Acid Res..

[27] Ni Z., Ye Y., Bal T., Xu M., Xu L.A. (2017). Complete chloroplast genome of *Pinus massoniana* (Pinaceae): Gene rearrangements, loss of ndh genes, and short inverted repeats contraction, expansion. Molecules.

[28] Hao D.C., Chen S.L., Huang B.L. (2009). Evolution of the chloroplast trnL-trnF region in the Gymnosperm lineages Taxaceae and Cephalotaxaceae. Biochem. Genet..

[29] Drescher A., Ruf S., Calsa T., Carrer H., Bock R. (2000). The two largest chloroplast genome-encoded open reading frames of higher plants are essential genes. Plant J..

[30] Park S., An B., Park S. (2018). Reconfiguration of the plastid genome in *Lamprocapnos spectabilis*: IR boundary shifting, inversion, and intraspecific variation. Sci. Rep..

[31] Barthet M.M., Hilu K.W. (2007). Expression of *matK*: Functional and evolutionary implications. Am. J. Bot..

[32] Arias S., Gama-López S., Guzmán-Cruz L.U., Vázquez-Benítez B., Medina L.R. (2012). Cactaceae. Flora del Valle de Tehuacán-Cuicatlán.

[33] Rozen S., Skaletsky H. (2000). Primer3 on the WWW for general users and for biologist programmers. Methods Mol. Biol..

[34] Dierckxsens N., Mardulyn P., Smits G. (2017). NOVOPlasty: De novo assembly of organelle genomes from whole genome data. Nucleic Acids Res..

[35] Love R.R., Weisenfeld N.I., Jaffe D.B., Besansky N.J., Neafsey D.E. (2016). Evaluation of DISCOVAR de novo using a mosquito simple for cost-effective short-read genome assembly. BMC Genom..

[36] Kolmogorov M., Raney B., Paten B., Pham S. (2014). Ragout-a reference-assisted assembly tool for bacterial genomes. Bioinformatics.

[37] Soto-Jiménez L.M., Estrada K., Sanchez-Flores A. (2014). GARM: Genome assembly, reconciliation and merging pipeline. Curr. Top. Med. Chem..

[38] Hunt M., De Sila N., Otto T.D., Parkhill J., Keane J.A., Harris S.R. (2015). Circlator: Automated circularization of genome assemblies using long sequencing reads. Genome Biol..

[39] Hunt M., Kikuchi T., Sanders M., Newbold C., Berriman M., Otto T.D. (2013). REAPR: A universal tool for genome assembly evaluation. Genome Biol..

[40] Tillich M., Lehwark P., Pellizzer T., Ulbricht-Jones E.S., Fischer A., Bock R., Greiner S. (2017). GeSeq–versatile and accurate annotation of organelle genomes. Nucleic Acids Res..

[41] Lohse M., Drechsel O., Bock R. (2007). OrganellarGenomeDRAW (OGDRAW): A tool for the easy generation of high-quality custom graphical maps of plastid and mitochondrial genomes. Curr. Genet..

[42] Liu X., Yang H., Zhao J., Zhou B., Li T., Xiang B. (2018). The complete chloroplast genome sequence of the folk medicinal and vegetable plant purslane (*Portulaca oleracea* L.). J. Hortic. Sci. Biotech..

[43] Darling A.C., Mau B., Blattner F.R., Perna N.T. (2004). Mauve: Multiple alignment of conserved genomic sequence with rearrangements. Genome Res..

[44] Darriba D., Taboada G.L., Doallo R., Posada D. (2011). ProtTest 3: Fast selection of best-fit models of protein evolution. Bioinformatics.

[45] Katoh K., Daron M.S. (2013). MAFFT multiple sequence alignment software version 7: Improvements in performance and usability. Mol. Biol. Evol..

[46] Posada D. (2008). JModelTest: Phylogenetic model averaging. Mol. Biol. Evol..

[47] Stamatakis A. (2014). RAxML version 8: A tool for phylogenetic analysis and post-analysis of large phylogenies. Bioinformatics.

