# Supplementary material for: De Novo Assembly Discovered Novel Structures in Genome of Plastids and Revealed Divergent Inverted Repeats in Mammillaria (Cactaceae, Caryophyllales)
Source: Plants (Basel). 2019 Oct 1;8(10):392. doi: 10.3390/plants8100392 (PMC6843559; doi:10.3390/plants8100392)
Supplement: Supplementary file 1 [file plants-08-00392-s001.zip › SuplementaryTables/SuplementayMaterialS2.588434.pdf]

Supplementary material

Table S2. List of the total of 42 coding loci were used to obtain phylogenetic relationships of the seven species of *Mammillaria* studied and the two species used as outgroups *Carnegiea gigantea and Portulaca oleraceae*.

# Species	Genes	Alg.-Conn.	*M_albiflora*	*M_crucigera*	*M_huitzilopochtli*	*M_pectinifera*	*M_solisoides*	*M_supertexta*	*M_zephyranthoides*	*Carnegiae_gigantea*	*Portulaca_oleracea*
9	9	0.94	01_psbJ	04_psbJ	06_psbJ	13_psbJ	15_psbJ	19_psbJ	23_psbJ	YP_009159769.1	YP_009441454.1
9	9	0.94	01_petD	04_petD	06_petD	13_petD	15_petD	19_petD	23_petD	YP_009159784.1	YP_009441471.1
9	9	0.939	01_rpoC2	04_rpoC2	06_rpoC2	13_rpoC2	15_rpoC2	19_rpoC2	23_rpoC2	YP_009159816.1	YP_009441430.1
9	9	0.938	01_rpoC1	04_rpoC1	06_rpoC1	13_rpoC1	15_rpoC1	19_rpoC1	23_rpoC1	YP_009159817.1	YP_009441431.1
9	9	0.938	01_rps4	04_rps4	06_rps4	13_rps4	15_rps4	19_rps4	23_rps4	YP_009159761.1	YP_009441442.1
9	9	0.93	01_atpE	04_atpE	06_atpE	13_atpE	15_atpE	19_atpE	23_atpE	YP_009159764.1	YP_009441446.1
9	9	0.924	01_cemA	04_cemA	06_cemA	13_cemA	15_cemA	19_cemA	23_cemA	YP_009159767.1	YP_009441452.1
9	9	0.921	01_rpl16	04_rpl16	06_rpl16	13_rpl16	15_rpl16	19_rpl16	23_rpl16	YP_009159791.1	YP_009441478.1
9	9	0.919	01_atpA	04_atpA	06_atpA	13_atpA	15_atpA	19_atpA	23_atpA	YP_009159811.1	YP_009441425.1
9	9	0.919	01_rpoA	04_rpoA	06_rpoA	13_rpoA	15_rpoA	19_rpoA	23_rpoA	YP_009159785.1	YP_009441472.1
9	9	0.918	01_psbI	04_psbI	06_psbI	13_psbI	15_psbI	19_psbI	23_psbI	YP_009159809.1	YP_009441424.1
9	9	0.917	01_atpI	04_atpI	06_atpI	13_atpI	15_atpI	19_atpI	23_atpI	YP_009159814.1	YP_009441428.1
9	9	0.917	01_atpB	04_atpB	06_atpB	13_atpB	15_atpB	19_atpB	23_atpB	YP_009159763.1	YP_009441447.1
9	9	0.916	01_matK	04_matK	06_matK	13_matK	15_matK	19_matK	23_matK	YP_009159806.1	YP_009441421.1
9	9	0.914	01_psaB	04_psaB	06_psaB	13_psaB	15_psaB	19_psaB	23_psaB	YP_009159825.1	YP_009441439.1
9	9	0.914	01_petA	04_petA	06_petA	13_petA	15_petA	19_petA	23_petA	YP_009159768.1	YP_009441453.1
9	9	0.913	01_psbA	04_psbA	06_psbA	13_psbA	15_psbA	19_psbA	23_psbA_b	YP_009159805.1	YP_009441420.1
9	9	0.912	01_petG	04_petG	06_petG	13_petG	15_petG	19_petG	23_petG	YP_009159774.1	YP_009441459.1
9	9	0.912	01_rbcL	04_rbcL	06_rbcL	13_rbcL	15_rbcL	19_rbcL	23_rbcL	YP_009159762.1	YP_009441448.1
9	9	0.911	01_psbK	04_psbK	06_psbK	13_psbK	15_psbK	19_psbK	23_psbK	YP_009159808.1	YP_009441423.1
9	9	0.91	01_psaC	04_psaC	06_psaC	13_psaC	15_psaC	19_psaC	23_psaC	YP_009159799.1	YP_009441491.1
9	9	0.91	01_psbE	04_psbE	06_psbE	13_psbE	15_psbE	19_psbE	23_psbE	YP_009159772.1	YP_009441457.1
9	9	0.91	01_psaA	04_psaA	06_psaA	13_psaA	15_psaA	19_psaA	23_psaA	YP_009159759.1	YP_009441440.1
9	9	0.909	01_psbL	04_psbL	06_psbL	13_psbL	15_psbL	19_psbL	23_psbL	YP_009159770.1	YP_009441455.1
9	9	0.908	01_atpH	04_atpH	06_atpH	13_atpH	15_atpH	19_atpH	23_atpH	YP_009159813.1	YP_009441427.1
9	9	0.907	01_psbB	04_psbB	06_psbB	13_psbB	15_psbB	19_psbB	23_psbB	YP_009159779.1	YP_009441466.1
9	9	0.906	01_psbC	04_psbC	06_psbC	13_psbC	15_psbC	19_psbC	23_psbC	YP_009159822.1	YP_009441436.1
9	9	0.904	01_rps8	04_rps8	06_rps8	13_rps8	15_rps8	19_rps8	23_rps8	YP_009159789.1	YP_009441476.1
9	9	0.903	01_rps15	04_rps15	06_rps15	13_rps15	15_rps15	19_rps15	23_rps15	YP_009159798.1	YP_009441497.1
9	9	0.902	01_psbD	04_psbD	06_psbD	13_psbD	15_psbD	19_psbD	23_psbD	YP_009159821.1	YP_009441435.1
9	9	0.9	01_petB	04_petB	06_petB	13_petB	15_petB	19_petB	23_petB	YP_009159783.1	YP_009441470.1
9	9	0.899	01_ccsA	04_ccsA	06_ccsA	13_ccsA	15_ccsA	19_ccsA	23_ccsA	YP_009159800.1	YP_009441489.1
9	9	0.899	01_petN	04_petN	06_petN	13_petN	15_petN	19_petN	23_petN	YP_009159819.1	YP_009441433.1
9	9	0.899	01_rps14	04_rps14	06_rps14	13_rps14	15_rps14	19_rps14	23_rps14	YP_009159824.1	YP_009441438.1
9	9	0.89	01_psbT	04_psbT	06_psbT	13_psbT	15_psbT	19_psbT	23_psbT	YP_009159780.1	YP_009441467.1
9	9	0.882	01_psbN	04_psbN	06_psbN	13_psbN	15_psbN	19_psbN	23_psbN	YP_009159781.1	YP_009441468.1

9	9	0.881	01_rpl14	04_rpl14	06_rpl14	13_rpl14	15_rpl14	19_rpl14	23_rpl14	YP_009159790.1		YP_009441477.1	
9	9	0.878	01_psbF	04_psbF	06_psbF	13_psbF	15_psbF	19_psbF	23_psbF	YP_009159771.1		YP_009441456.1	
9	9	0.87	01_rps2	04_rps2	06_rps2	13_rps2	15_rps2	19_rps2	23_rps2	YP_009159815.1		YP_009441429.1	
9	9	0.866	01_petL	04_petL	06_petL	13_petL	15_petL	19_petL	23_petL	YP_009159773.1		YP_009441458.1	
9	9	0.841	01_rps3	04_rps3	06_rps3	13_rps3	15_rps3	19_rps3	23_rps3	YP_009159792.1		YP_009441479.1	
9	9	0.838	01_psbH	04_psbH	06_psbH	13_psbH	15_psbH	19_psbH	23_psbH	YP_009159782.1		YP_009441469.1	
9	9	0.766	01_rps11	04_rps11	06_rps11	13_rps11	15_rps11	19_rps11	23_rps11	YP_009159786.1		YP_009441473.1	
9	9	0.698	01_infA	04_infA	06_infA	13_infA	15_infA	19_infA	23_infA	YP_009159788.1		YP_009441475.1	
9	9	0.693	01_rpl20	04_rpl20	06_rpl20	13_rpl20	15_rpl20	19_rpl20	23_rpl20	YP_009159777.1		YP_009441463.1	
9	9	0.663	01_rps19	04_rps19	06_rps19	13_rps19	15_rps19	19_rps19	23_rps19	YP_009159794.1		YP_009441481.1	
9	9	0.65	01_rpoB	04_rpoB	06_rpoB	13_rpoB	15_rpoB	19_rpoB	23_rpoB	YP_009159818.1		YP_009441432.1	
9	9	0.45	01_rpl22	04_rpl22	06_rpl22	13_rpl22	15_rpl22	19_rpl22	23_rpl22	YP_009159793.1		YP_009441480.1	
9	10	0.763	01_rpl2	04_rpl2	06_rpl2	13_rpl2	15_rpl2	19_rpl2	23_rpl2	YP_009159795.1		YP_009441482.1,YP_009441503.1	
9	10	0.705	01_rps7	04_rps7	06_rps7	13_rps7	15_rps7	19_rps7	23_rps7	YP_009159804.1		YP_009441486.1,YP_009441499.1	
8	8	0.913	01_psbZ	04_psbZ	06_psbZ	13_psbZ	15_psbZ	19_psbZ	*	YP_009159823.1		YP_009441437.1	
8	8	0.906	*	04_psaI	06_psaI	13_psaI	15_psaI	19_psaI	23_psaI	YP_009159765.1		YP_009441450.1	
8	8	0.714	01_rpl32	04_rpl32	06_rpl32	13_rpl32	15_rpl32	19_rpl32	23_rpl32_a		YP_009159801.1		*
7	7	0.385	01_psbM	04_psbM	*	13_psbM	15_psbM	*	23_psbM	YP_009159820.1		YP_009441434.1	
6	6	0.94	*	04_psaJ	06_psaJ	13_psaJ	15_psaJ	19_psaJ	23_psaJ	*	*		
3	3	0.946	01_psaJ	*	*	*	*	*	*	YP_009159775.1		YP_009441460.1	
2	3	0.333	*	*	*	*	*	*	*	YP_009159796.1		YP_009441484.1,YP_009441501.1	
2	3	0.333	*	*	*	*	*	*	*	YP_009159778.1		YP_009441419.1,YP_009441464.1	
2	2	1	*	*	*	*	*	*	*	YP_009159812.1		YP_009441426.1	
2	2	1	*	*	*	*	*	*	*	YP_009159810.1		YP_009441465.1	
2	2	1	*	*	*	*	*	*	*	YP_009159807.1		YP_009441422.1	
2	2	1	*	*	*	*	*	*	*	YP_009159797.1		YP_009441498.1	
2	2	1	*	*	06_psbM	*	*	19_psbM	*	*	*		
2	2	1	*	*	*	*	*	*	*	YP_009159776.1		YP_009441462.1	
2	2	1	*	*	*	*	*	*	*	YP_009159766.1		YP_009441451.1	
2	2	1	*	*	*	*	*	*	*	YP_009159760.1		YP_009441441.1	
2	2	1	*	*	*	*	*	*	*	YP_009159787.1		YP_009441474.1	
