# Supplementary material for: De Novo Assembly Discovered Novel Structures in Genome of Plastids and Revealed Divergent Inverted Repeats in Mammillaria (Cactaceae, Caryophyllales)
Source: Plants (Basel). 2019 Oct 1;8(10):392. doi: 10.3390/plants8100392 (PMC6843559; doi:10.3390/plants8100392)

Supplementary Material

Table S1. Primer sequences designed for PCR verifications for flanking adjacent sequences of IRs; and gene rearrangements located at LSC in three genomes structures of *Mammillaria.* In the last column the temperature used to amplify each locus for all species. Primer design was based on the DNA sequences of *M. albiflora* (structure 1), *M. supertexta* (structure 2) and *M. zephyranthoides* (structure 3).

Locus name	Primer Sequence (5′-)	Structure identified	T (°C)
rpls19-rps16	Frps19 CGGTGCAAATTCTCCCAATT<br>Rrps16 CGTTGCTTTCTACCACATCG	This locus amplifies only if the flanking of IRB has rps19-trnQ (Structure 2)	57
rpl2-ycf2	3Frpl2 TCCCCTCTTTTGCAATCAGT<br>3Rycf2 AAGCGCCCAACTCATAATTG	This locus amplifies for IRB of the three structures; however, the PCR product has nearly a double size for structure 3, because it has psbA inserted in IRB.	57
ycf2-rpl23	11F TGTTGATACAAAGCAGCCA<br>11F ACCCGATTGAAGCGTAATGA	This locus amplifies the ycf2-rpl23 of IRB in three structures. It was not expected to amplify in structure 3 but the Sanger sequencing discovered rpl23 in its IRB.	56
ndhB-rpl2	9F TTGGAGATTGGATGCGTTA<br>9R ACGTCTTGTGGGATAGCATT	This locus amplifies flanking and the end part of IRB of Structure 2. It amplifies a smallest product for Structure 1 and it does not amplify for Structure 3.	57
psbA-trnK	FpsbAzep TGGAGGAGCAGCAATGAAGG<br>RtrnKzep TGTGCTAGAACTTTGGCTCGT	This locus amplifies the flanking of IRA of structure 3.	60
trnP-rpl20	FMzeptrnP GTAGCGCGTTTGTTTTGG<br>RMzeprpl20<br>TGACTAGAGTTAGACGGGGATA	This locus amplifies only if the gene arrangement of trnP-rpl20 of sructure 3 is	58
trnS-psbZ	30F TACCTTGACACCARAGAGRG<br>30RCACCGCTTCCACAGCTTTCT	This locus only amplifies for structure 3, or unspecific PCR product of nearly 300 bp may be obtained for Structures 1 and 2, which are eliminated by increasing the alignment temperature.	57

Supplementary material (continued). Examples of PCR verifications carried out to test gene rearrangements found in seven species of *Mammillaria.* Standard PCR cycle was carried consisted of initial denaturation at 95°C-3 min, and 34 cycles included denaturing at 95 °C -45 sec, primer alignment (57-60 °C)-40″, synthesis at 72°C –1.15 min; and final extension at 72°C -5 min. In all photos are presented PCR results run in agarose gels 1.5% stained with Syber Safe and visualized with UV light, and the size was referred to 1 Kb Plus DNA ladder with smallest line is of

100 bp. In all gels the samples were loaded in lanes in alphabetical order from left to the right: *M. albiflora, M. crucigera, M. huitzilopochtli, M. pectinifera, M. solisioides, M. supertexta* and *M. zephyranthoides.*

a) Gene rearrangement of psbZ-trnS. The pair of primers 30F/30F only amplifies the segment of psbZ-trnS with the orientation recorded in the structure 3. The unspecific weak products were eliminated by increasing 1-2 °C s the alignment primers temperature.

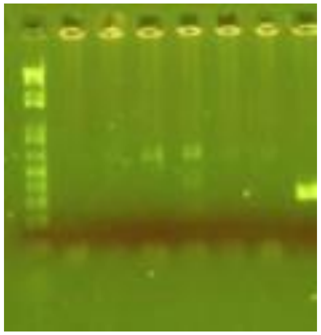

b) To the right of the gel the PCR obtained with the pair of primers 9F/9R (locus ndhB-rpl2) from lanes numbered from 50-56. In structure 3 this locus does not amplified, since these genes have not the same order to that found in structures 1 and 2. The left gel is placed in order to have the DNA ladder size.

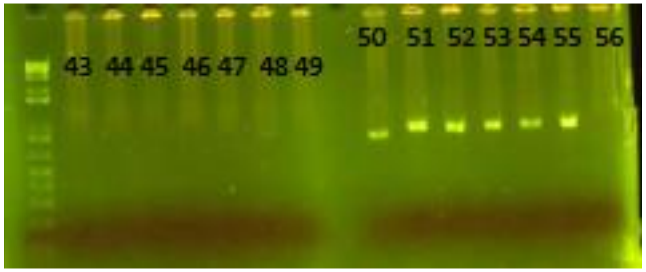

c) PCR amplification obtained for locus 3F/3R, which amplified rpl23-ycf2 of IRB for seven species, but in species with structure 3 it has a heavier band because it has inserted psbA at IRB.

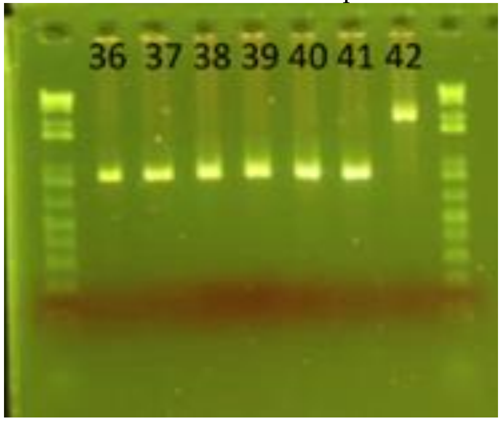

d) PCR result to test the locus rps19-rps16 of IRB of structure 2, which is absent in species with structure 1 and structure 3, and consequently this locus did not amplify.

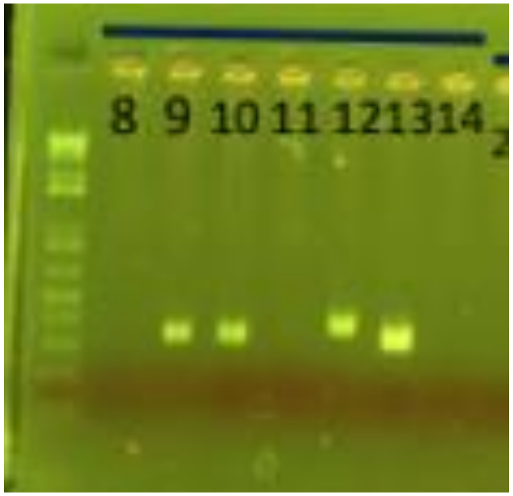

Supplement: Supplementary file 1 [file plants-08-00392-s001.zip › SuplementaryTables/SupplementaryMaterialS1.588434.pdf]
