# Supplementary material for: De Novo Assembly Discovered Novel Structures in Genome of Plastids and Revealed Divergent Inverted Repeats in Mammillaria (Cactaceae, Caryophyllales)
Source: Plants (Basel). 2019 Oct 1;8(10):392. doi: 10.3390/plants8100392 (PMC6843559; doi:10.3390/plants8100392)
Supplement: Supplementary file 1 [file plants-08-00392-s001.zip › SuplementaryTables/SupplementaryMaterialS1.588434.doc]

***Supplementary Material***

**Table S1.** Primer sequences designed for PCR verifications for flanking adjacent sequences of IRs; and gene rearrangements located at LSC in three genomes structures of *Mammillaria.* In the last column the temperature used to amplify each locus for all species. Primer design was based on the DNA sequences of *M. albiflora* (structure 1), *M. supertexta* (structure 2) and *M. zephyranthoides* (structure 3).

| Locus name | Primer Sequence (5’-) | Structure identified | T (°C) |
| --- | --- | --- | --- |
| rpls19-rps16 | Frps19 CGGTGCAAATTCTCCCAATT  Rrps16 CGTTGCTTTCTACCACATCG | This locus amplifies only if the flanking of IRB has rps19-trnQ (Structure 2) | 57 |
| rpl2-ycf2 | 3Frpl2 TCCCCTCTTTTGCAATCAGT  3Rycf2 AAGCGCCCAACTCATAATTG | This locus amplifies for IRB of the three structures; however, the PCR product has nearly a double size for structure 3, because it has psbA inserted in IRB. | 57 |
| ycf2-rpl23 | 11F TGTTGATACAAAGCAGCCA  11F ACCCGATTGAAGCGTAATGA | This locus amplifies the ycf2-rpl23 of IRB in three structures. It was not expected to amplify in structure 3 but the Sanger sequencing discovered rpl23 in its IRB. | 56 |
| ndhB-rpl2 | 9F TTGGAGATTGGATGCGTTA  9R ACGTCTTGTGGGATAGCATT | This locus amplifies flanking and the end part of IRB of Structure 2. It amplifies a smallest product for Structure 1 and it does not amplify for Structure 3. | 57 |
| psbA-trnK | FpsbAzep TGGAGGAGCAGCAATGAAGG  RtrnKzep TGTGCTAGAACTTTGGCTCGT | This locus amplifies the flanking of IRA of structure 3. | 60 |
| trnP-rpl20 | FMzeptrnP GTAGCGCGTTTGTTTTGG  RMzeprpl20 TGACTAGAGTTAGACGGGGATA | This locus amplifies only if the gene arrangement of trnP-rpl20 of sructure 3 is | 58 |
| trnS-psbZ | 30F TACCTTGACACCARAGAGRG  30RCACCGCTTCCACAGCTTTCT | This locus only amplifies for structure 3, or unspecific PCR product of nearly 300 bp may be obtained for Structures 1 and 2, which are eliminated by increasing the alignment temperature. | 57 |

**Supplementary material (continued**). Examples of PCR verifications carried out to test gene rearrangements found in seven species of *Mammillaria.* Standard PCR cycle was carried consisted of initial denaturation at 95°C-3 min, and 34 cycles included denaturing at 95 °C -45 sec, primer alignment (57-60 °C)-40”, synthesis at 72°C –1.15 min; and final extension at 72°C -5 min. In all photos are presented PCR results run in agarose gels 1.5% stained with Syber Safe and visualized with UV light, and the size was referred to 1 Kb Plus DNA ladder with smallest line is of 100 bp. In all gels the samples were loaded in lanes in alphabetical order from left to the right: *M. albiflora, M. crucigera, M. huitzilopochtli, M. pectinifera, M. solisioides, M. supertexta* and *M. zephyranthoides.*

a) Gene rearrangement of psbZ-trnS. The pair of primers 30F/30F only amplifies the segment of psbZ-trnS with the orientation recorded in the structure 3. The unspecific weak products were eliminated by increasing 1-2 °C s the alignment primers temperature.

b) To the right of the gel the PCR obtained with the pair of primers 9F/9R (locus ndhB-rpl2) from lanes numbered from 50-56. In structure 3 this locus does not amplified, since these genes have not the same order to that found in structures 1 and 2. The left gel is placed in order to have the DNA ladder size.

c) PCR amplification obtained for locus 3F/3R, which amplified rpl23-ycf2 of IRB for seven species, but in species with structure 3 it has a heavier band because it has inserted psbA at IRB.

d) PCR result to test the locus rps19-rps16 of IRB of structure 2, which is absent in species with structure 1 and structure 3, and consequently this locus did not amplify.
